# Applied research on the design of protective clothing based on the Kano-QFD-PUGH method

**DOI:** 10.1371/journal.pone.0312045

**Published:** 2024-10-24

**Authors:** Hong Li, Li Shi

**Affiliations:** 1 Guangzhou Huashang College, Guangzhou, China; 2 Faculty of Innovation and Design, City University of Macau, Macau, China; Southern Taiwan University of Science and Technology, TAIWAN

## Abstract

In order to improve the user experience of protective clothing for healthcare workers and reduce the design blindness and subjectivity of developers, we propose a research methodology that combines the Kano model, QFD quality function deployment, and PUGH decision-making scheme to develop conceptual solutions for medical protective clothing design. Firstly, we use the Kano model to identify the user requirements of healthcare workers and construct a hierarchy of functional requirements for protective clothing. Secondly, we use the QFD method to weigh the protective clothing design elements, convert user requirements into design elements, establish a relationship matrix between user requirements and design elements, and generate four conceptual design solutions based on the results. Finally, we use the PUGH decision-making method to filter and select the best concept solution for protective clothing design, and validate the design evaluation. Our results show that the protective clothing solutions designed using the combined Kano-QFD-PUGH system approach have a higher level of satisfaction compared to traditional protective clothing design. This method accurately explores the mapping relationship between user requirements and design functional elements and can be used as a general reliability design method. It helps to improve the development efficiency of designers and the decision-making role for design concept solution preference. Overall, our research methodology provides a comprehensive approach to developing medical protective clothing, which can be useful for designers and decision-makers in the healthcare industry.

## Introduction

With the outbreak of the new crown epidemic around the world, medical protective clothing has become an important protective equipment tool in the fight against the epidemic. Much attention has been paid to the design of protective clothing, not only for the protection of healthcare workers but also for the safety of the general population [1]. However, during the COVID-19 epidemic, the user experience of protective clothing was unsatisfactory and there were many problems with the design of protective clothing. One of the most important measures to prevent the spread of the virus was the wearing of masks and protective clothing, as people’s habits changed dramatically in the wake of the COVID-19 outbreak. Protective clothing is more than just a garment; it is more of a social responsibility, a shared responsibility of each individual to act to protect themselves and others. In the face of the possibility of another relevant public health event in the future, the problems associated with protective clothing in the current epidemic are analyzed and a method of converting user needs into functional elements of design is proposed [2], thus improving the development and design of protective clothing and, indirectly, the ability of society as a whole to prevent epidemics and resist risk. Therefore, it is particularly important to study the design and development system of protective clothing in the post-epidemic era and to provide some theoretical value for the construction of a standardized system of protective clothing [3].

The forms and uses of protective garments have been enhanced and honed over time thanks to technological advancements and a focus on health. Medical protective apparel was widely available during the COVID-19 outbreak and was successful in blocking the penetration of alcohol, body fluids, aerosol powder, dust, and bacteria. The major components of this protective gear were a cap, top, and pants. It had a one-piece structure that made it simple to wear and had pieces that were very firmly connected, substantially enhancing its protective effectiveness. The management of epidemics has become more complex due to virus mutation, and this has caused several problems with protective apparel, including its sole purpose, irrational pattern, poor breathability, and weak identification [4].

As an important piece of protective equipment in epidemic prevention work, the problems that existed in protective clothing during this epidemic must be confronted and will directly affect the experience and efficiency of future epidemic prevention personnel [5]. The design of protective clothing is a complex system project that must take into account a variety of application scenario factors to ensure the protective performance of the clothing. In order to ensure a good experience of protective clothing, the design process must take into account user needs, the relationship between functional elements and the trade-offs between design factors. This requires a scientific system to be used to build the protective clothing design process so as to ensure that protective clothing development is scientific and efficient [6]. Therefore, a scientific system process for the design and development of protective clothing in response to public health events must be constructed. This study proposes a comprehensive solution for the development of protective clothing design using the Kano model, combined with QFD (Quality Function Deployment), and the PUGH Decision Matrix. This method is different from the traditional design method which is based on the designer’s subjective consciousness, and helps designers and developers to design protective clothing scientifically and accurately, and to establish a scientific design method and process system for future epidemic prevention applications.

## Methods

### Overview of the Kano-QFD-PUGH method

The Kano model is a tool used to identify and analyze personalized user needs, primarily employing qualitative methods to categorize these needs. It plays a critical role during the product research phase by mapping user satisfaction feedback to product functionalities, thus enabling an effective prioritization of requirements [7]. In contrast to the qualitative analysis used in the Kano model, QFD (Quality Function Deployment) applies quantitative methods to study the relationship between user needs and product characteristics. By using matrix analysis, QFD transforms user needs into specific functional requirements and quantifies the priority of these functions [8]. This allows QFD to further refine the needs identified by the Kano model, helping design teams clearly translate user demands into practical design plans and organize development tasks based on priority. QFD plays a pivotal role in converting user needs into functional requirements.

The PUGH decision matrix, on the other hand, is a tool used to compare and select design options. It scores and compares alternative solutions against a reference, helping teams make the most logical choices among multiple options. The PUGH matrix is particularly useful in the later stages of product design, focusing on functional selection and design optimization [9]. It uses the core needs identified by the Kano model and the functional requirements analyzed through QFD to compare the advantages and disadvantages of different design schemes. This enables design teams to make more logical and well-reasoned decisions in complex scenarios. Ultimately, the three methods work together to form a complete product development and design process.

In the existing literature, the application of Kano, QFD, and PUGH has been widely discussed across various fields. Fang et al. proposed an integrated KANO-AHP-QFD-PUGH model for the design of a medication reminder app for the elderly, demonstrating how the method not only optimizes user experience but also fosters innovation and sustainability in design [10]. This study shows how effectively user needs and technical solutions can be integrated. Deng Xinyi et al. combined the Kano-QFD model with user journey mapping to optimize a fitness game system, improving user interaction. The strength of this combined model lies in its ability to comprehensively capture user needs and translate them into functional design improvements [11]. Gangurde applied the integration of Kano and QFD to explore its practical application in mobile phone products, providing a basis for product development decisions [12]. This method showcases how prioritizing customer needs can enhance user satisfaction. Additionally, Li Hui et al. introduced a QFD and PUGH combined evaluation model, improving the evaluation process for human-machine interfaces by incorporating the Analytical Hierarchy Process (AHP), significantly enhancing the design quality of interactive systems [13]. Zhou Hongyu et al. proposed a product design and development process based on the Kano-QFD-PUGH model, which effectively addresses the issue of compatibility between laser cleaning machines and specialized equipment [14]. However, this method primarily focuses on functional compatibility and safety in industrial applications, adhering to relatively traditional design concepts, and lacks innovative consideration of user comfort and environmental factors.

In summary, although existing research has extensively explored the integrated application of Kano, QFD, and PUGH, a unified research paradigm has yet to emerge. Applications of these methods often require customization based on specific contexts. My study, “Applied Research on the Design of Protective Clothing Based on the Kano-QFD-PUGH Method,” will further expand the application of these three methods in the field of functional clothing design. By combining user needs with the functional design of protective clothing, this research aims to provide more precise demand identification and functional optimization strategies, addressing the inadequacies in the conversion of user needs in current product design.

### Introduction to the Kano-QFD-PUGH model

According to an analysis of the impact of user needs on user satisfaction, Professor Noriaki Kano of the Tokyo Institute of Technology created the KANO model, a tool for categorizing and prioritizing user needs that captures the non-linear relationship between product performance and user satisfaction [15]. User happiness and product performance have a non-linear connection. The tool’s primary goal is to display the relationship between customer happiness and product performance. Five categories of user demands are distinguished: basic, wanted, attractive, undifferentiated, and reversal.

User demands are converted into design requirements using QFD models. Design specifications for epidemic-proof apparel include material choice, structural design, functional design, and other elements. The significance and priority of various design requirements can be established by comparing user needs with design requirements [16]. A customer needs satisfaction table, a design factor relationship table, and a design factor trade-off table make up the three components of the QFD technique. In the process of designing anti-epidemic clothes, the QFD technique can assist us in converting consumer requests into design considerations. In order to determine the design factors for protective clothing, we can first create a customer needs satisfaction table, then a design factor relationship table to determine the relationships between the design factors, and finally a design factor trade-off table to determine the design concept solutions for protective clothing [17].

The PUGH approach is a way of weighing several elements to assist in weighing design factors and coming up with a specific design for protective garments. The PUGH approach is composed of three steps: screening potential design factors, evaluating potential design elements, and weighting potential design considerations to select the optimum design solution. The PUGH model is employed to assess the benefits and drawbacks of various design alternatives. The PUGH model is employed to assess the benefits and drawbacks of various design alternatives [18]. Different materials, different structures, different functions, and other possibilities are available when designing protective apparel. The best protective garment design can be established by weighing the many design possibilities.

### Research process based on Kano-QFD-PUGH protective clothing design

**Fig 1:**
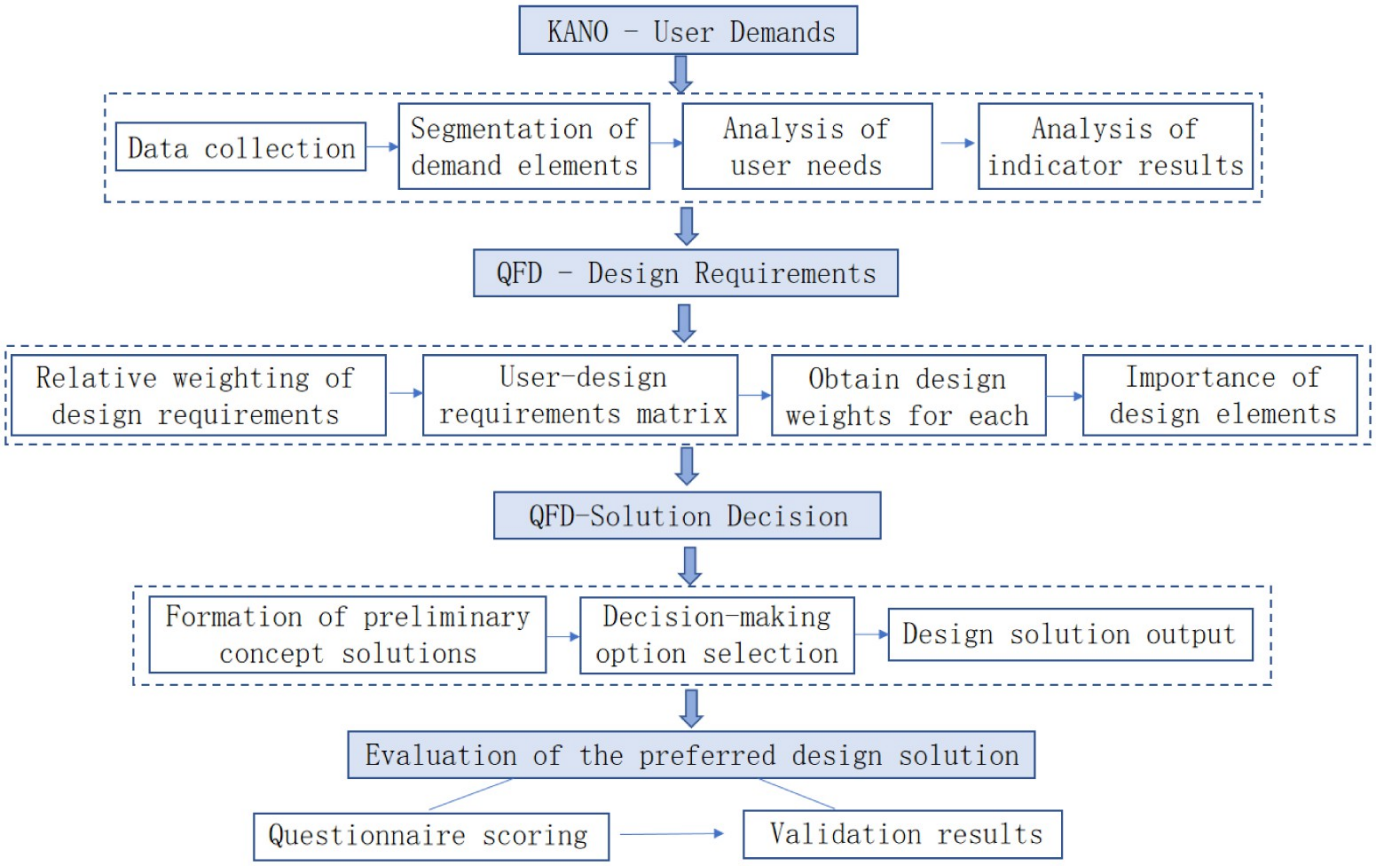
Protective clothing design development process based on the Kano-QFD-PUGH method.

In the design of protective clothing, the Kano-QFD-PUGH method can be used to identify user needs, translate them into design requirements and evaluate the advantages and disadvantages of different design solutions, thus improving the quality and effectiveness of protective clothing. the research application of the Kano-QFD-PUGH method in the protective clothing design process system (shown in Fig 1). Combined with the protective clothing design characteristics of this design project, the global phase planning is carried out and the specific research step process is as follows.

### Step 1: Utilizing the Kano model to extract user requirements

Through surveys and user interviews, the needs of different healthcare personnel regarding the safety, comfort, environmental friendliness, and operability of protective clothing are identified. Based on the collected data, the Kano model is used to classify these needs into basic, performance, excitement, indifferent, and reverse categories [19]. By calculating the importance of these needs, they are ranked to highlight key requirements. A combination of quantitative and qualitative methods is then used to calculate the importance of different needs, providing foundational support for constructing the quality house.

### Step 2: Using QFD to determine design elements

The user needs categorized by the Kano model are translated into specific design elements, including structural and functional design aspects of protective clothing. These design requirements are categorized and organized into a design function elements matrix, determining the importance and priority of each design element. The relationships between design elements are established by creating a design elements relationship matrix, allowing for a clear understanding of their interactions during the design process. Based on these design elements, different protective clothing design schemes, including various materials, structures, and functions, are determined [20]. These schemes are categorized and organized into a design scheme matrix, with the importance of design function elements ranked accordingly.

### Step 3: Evaluating schemes with the PUGH method

Initially, potential design factors that meet the design requirements are screened. The PUGH decision matrix is used to evaluate different protective clothing concept designs, with expert evaluations scoring each scheme against a benchmark. Finally, weights are calculated to determine the optimal design scheme.

### Step 4: Evaluating the selected optimal design scheme

Based on the PUGH evaluation results, the optimal protective clothing design scheme is optimized and improved. The traditional design is compared with the new method’s design scheme. User satisfaction is surveyed through questionnaires, evaluating visibility, functionality, ease of use, advancement, portability, comfort, and innovation [21]. The final validation is conducted to confirm the effectiveness of the protective clothing designed using the Kano-QFD-PUGH method.

In summary, Fig 1 details the specific analysis process from the Kano model to QFD and then to the PUGH decision evaluation. Through the systematic integration of these three steps, user needs are scientifically translated into design elements. This process, combined with a scientific comparison and trade-off mechanism, ensures the objectivity and scientific validity of design decisions, thereby improving the quality of protective clothing design and user satisfaction. This detailed process illustrates the entire workflow from need analysis to final design decision.

### User requirements analysis of protective clothing based on the Kano model

The Kano model research phase is divided into three main steps: initially, the gathering of basic data using the KJ method (Affinity Diagram) for the primary classification and screening of the design requirements of the preliminary research, the summary of the functional requirements of protective clothing as a research sample [22]; next, the creation of the KANO model requirements questionnaire, with the KANO questionnaire to define the design requirements at each stage of the research process; and in third place the implementation of the KANO model requirements questionnaire [23]. The Better-Worse coefficient analysis method was then employed to optimize the design of protective clothing by calculating the coefficients of each design requirement item and category, determining the weight rating, and creating a hierarchy of functional requirements for protective clothing.

### Basic data collection

This study included doctors, nurses, volunteers, and other individuals who engaged in the epidemic prevention work as the target group in order to make the survey more accurate. The project team investigated the locations for epidemic prevention and gathered data from those in charge of it. Firstly, in-depth interviews and on-site observations were used to collect and record users’ needs about protective clothing; secondly, the users’ descriptions of primitive functions were transformed into clear user need attributes [24]; finally, through the principle of function card classification in the KJ method (Affinity Diagram), the relevant need attributes were classified into categories and function indicators, and repetitive needs and invalid functions were removed elements, and primary and secondary indicators were set for the induction of protective clothing functional elements, which eventually formed a primary functional requirements list. (As shown in Table 1).

**Table 1:** Functional requirements analysis table (Source: Adapted from Han Wei et al., 2020).

Category	Serial number	Functional indicators	Category	Serial number	Functional indicators
**Security needs**	C1	Anti-bacterial function	**Comfort needs**	C13	Material softness
	C2	Anti-spittle function		C14	Comfortable in contact with the body
	C3	Waterproof function		C15	Does not interfere with sight lines
	C4	Anti-infection function		C16	Adjustable wear size
**Performance requirements**	C5	Dirt resistance	**Identifying needs**	C17	Clearly distinguish the front and back of your clothes
	C6	Airtight fit to the face		C18	Highly recognisable to distinguish users
	C7	Lightweight design		C19	Reasonable colour differentiation
	C8	Suitable for different seasons		C20	Distinguish between male and female models
**Environmental needs**	C9	Easy recycling and disposal	**Operational requirements**	C21	Ease of donning and doffing
	C10	Material degradability		C22	Smoothness of operation
	C11	Easy transport		C23	Convenience of storing objects
	C12	Modular functionality saves resources		C24	Convenient storage for carrying items

### Questionnaire design

In order to determine user demands, this study tackles the present aspects of protective clothing demand in China and suggests designing the questionnaire in two directions: functional items and human-machine interaction. The research questionnaire is broken into three sections [25]. The first section is a description, which provides a succinct explanation of the study’s objectives. The second section is a simple survey that looks into the user’s gender, occupation, prior use of protective gear, as well as the occasion and setting for using it. The third section is for the scale element, which includes the questionnaire’s questions and feelings [26]. Five options are provided for each question on a five-point Likert scale in this section(As shown in Table 2).From The questions and feelings section makes up the third section of the questionnaire. It uses a five-point Likert scale to offer five options for each question, collecting the user’s attitude toward both positive and negative aspects, meeting their needs and not meeting them, and includes five options for each question: very satisfied, satisfied, indifferent, dissatisfied, and very dissatisfied. This allows the user to select the option that best expresses how they are feeling [27].

**Table 2:** Five-point Likert scale (Source: Adapted from Luo Zheng qing et al., 2002).

	Demand item options				
**Provide this requirement**	Very satisfied	Satisfaction	Doesn’t matter	Not satisfied	Very dissatisfied
**Not available for this requirement**	Very satisfied	Satisfaction	Doesn’t matter	Not satisfied	Very unsatisfactory

### Questionnaire collection and testing

The questionnaires were distributed to those involved in epidemic prevention, i.e. doctors, nurses, epidemic prevention volunteers, medical cleaning staff, etc. A total of 146 questionnaires were distributed. A total of 128 questionnaires were returned and 19 invalid questionnaires were excluded to obtain 109 valid questionnaires. The KANO evaluation form was designed for the KANO model before processing the obtained user requirement data [28]. According to the KANO model functional indicators needs can be divided into five categories: basic needs (M), expectation needs (O), excitement needs (A), undifferentiated needs (I), and reverse needs (R). The correspondence between positive and negative questions and requirements is shown in Table 3. Prior to the start of the study, we obtained verbal informed consent from all participants indicating their willingness to fill out the questionnaire. The content of this study complies with the relevant ethical requirements of the Academic Committee of the School of Creativity and Design of Guangzhou Huashang University and has been approved by them, so it is exempted from the requirements of ethical review.

**Table 3:** KANO evaluation table (Source: Adapted from Kano et al., 1984).

User requirements		Reverse problem				
		Very satisfied	Satisfaction	Doesn’t matter	Not satisfied	Very dissatisfied
**Positive issues**	Very satisfied	Q	A	A	A	O
	Satisfaction	R	I	I	I	M
	Doesn’t matter	R	I	I	I	M
	Not satisfied	R	I	I	I	M
	Very dissatisfied	R	R	R	R	Q

Based on the data from this research, the questionnaire was analysed for reliability through SPSS22.0 statistical software, and the Cronbach’s alpha value for the positive questions was 0.805 and the Cronbach’s alpha value for the negative questions was 0.812, indicating that the research questionnaire had good internal consistency and the findings were credible. The results of the questionnaire were used to classify the demand attributes against the KANO evaluation scale [29], resulting in a summary of the KANO classification of protective clothing design demands, as shown in Table 4.

**Table 4:** Classification of design requirements KANO attributes (Source: Author’s own research).

Serial number	A	O	M	I	R	KANO properties
C1	8	28	73	0	0	M
C2	17	11	81	0	0	M
C3	26	38	24	6	0	O
C4	12	25	72	0	0	M
C5	17	26	64	2	0	M
C6	20	22	27	31	9	I
C7	27	48	23	11	0	O
C8	52	23	27	5	2	A
C9	30	45	31	3	0	O
C10	29	24	22	32	2	I
C11	16	26	21	39	7	I
C12	43	32	34	0	0	A
C13	37	30	35	7	0	A
C14	31	25	51	2	0	M
C15	19	21	69	0	0	M
C16	39	54	11	5	0	O
C17	25	22	62	0	0	M
C18	29	46	32	2	0	O
C19	53	23	27	6	0	A
C20	24	28	17	35	5	I
C21	38	33	30	8	0	A
C22	22	27	49	11	0	M
C23	42	38	24	5	0	A
C24	56	25	21	7	0	A

### Analysis of the results of user satisfaction indicators

Based on the data aggregated from the design requirements attributes of the KANO model, the results were calculated using the "customer satisfaction coefficient (CS)" proposed by C. Berger et al, i.e. the Better-Worse index analysis method. Better indicates the extent to which quality factors affect satisfaction [30], and the larger the positive value, the better the effect of customer satisfaction; Worse indicates the extent to which quality factors affect customer dissatisfaction, and the larger the negative value, the lower the customer satisfaction. The Better-Worse coefficient for the functional requirements of protective clothing can be calculated using the following formula.


$$\left\{\begin{array}{l} S_{i}=\frac{A_{i}+O_{i}}{A_{i}+O_{i}+M_{i}+I_{i}}\\ D_{i}=(-1)\times \frac{M_{i}+O_{i}}{A_{i}+O_{i}+M_{i}+I_{i}} \end{array}\right.$$

Where *S*_*i*_ represents the Better coefficient indicating the user’s satisfaction with the first i function, and *D*_*i*_ represents the Worse coefficient, which indicates the user’s dissatisfaction with the i The Better coefficient represents the coefficient of user satisfaction with the demand for function. The coefficients of *A*_*i*_, *O*_*i*_, *M*_*i*_ and *I*_*i*_ denotes the percentage of users’ demand choices in the questionnaire survey for each function in categories A, O, M and I, respectively [31].

Based on the above formula, the values of the 24 design requirement items were brought into the calculation to obtain the results shown in Table 5.

**Table 5:** Results of the Better-Worse index analysis for each indicator (Source: Author’s own research).

Serial number	Better	Worse	Serial number	Better	Worse
C1	0.33	-0.92	C13	0.61	-0.60
C2	0.26	-0.84	C14	0.51	-0.70
C3	0.59	-0.57	C15	0.37	-0.83
C4	0.34	-0.89	C16	0.85	-0.83
C5	0.39	-0.83	C17	0.43	-0.77
C6	0.42	-0.49	C18	0.69	-0.72
C7	0.69	-0.65	C19	0.70	-0.46
C8	0.70	-0.47	C20	0.50	-0.43
C9	0.69	-0.70	C21	0.65	-0.35
C10	0.50	-0.43	C22	0.45	-0.58
C11	0.41	-0.46	C23	0.73	-0.57
C12	0.69	-0.60	C24	0.74	-0.42

**Fig 2:**
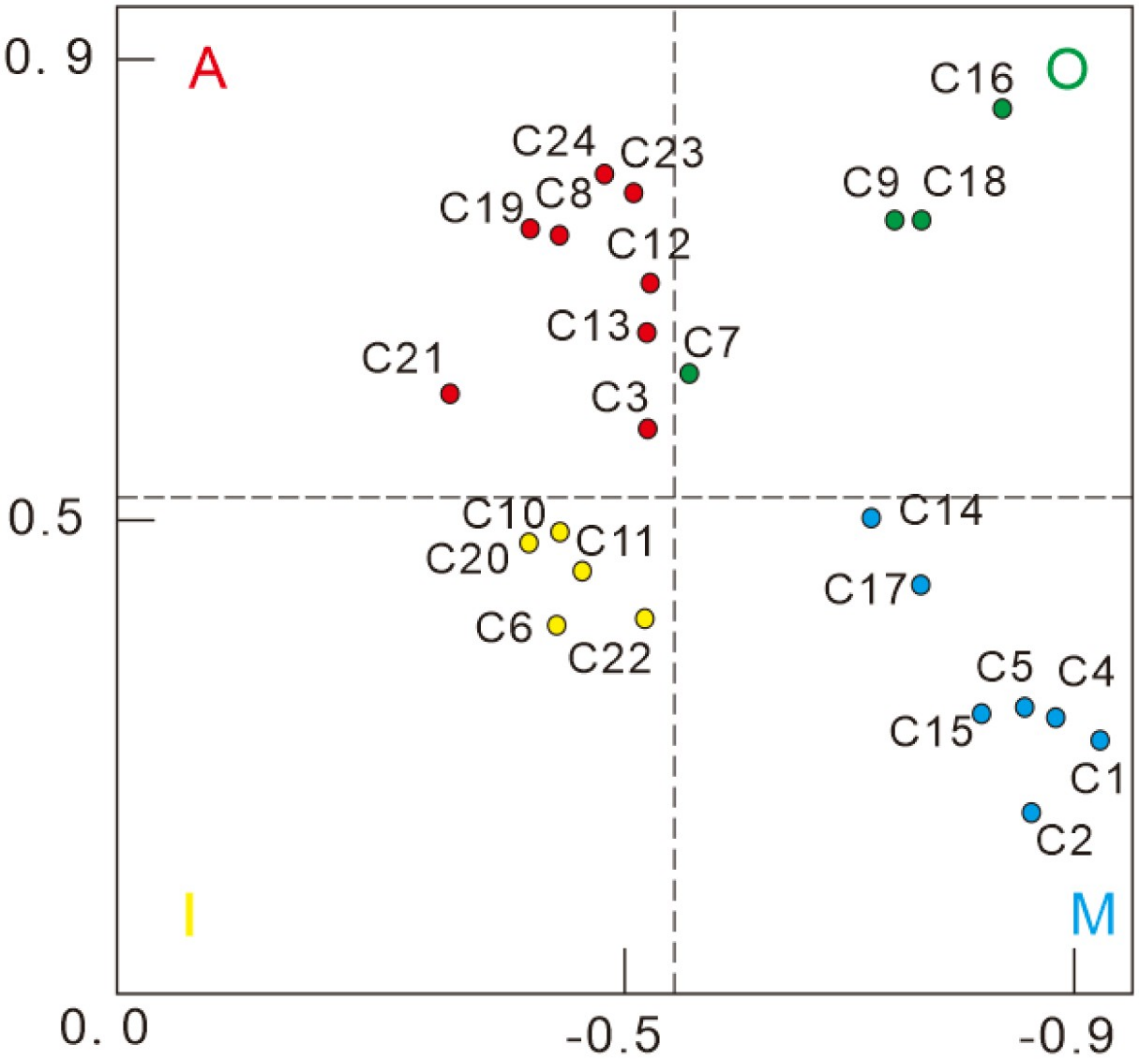
Four quadrant diagram. (A: Exciting demand; O: Desired demand; I: Non-differentiated needs; M: Basic needs).

The Better and Worse index values of each indicator are displayed on a four-quadrant diagram, with the Better value acting as the horizontal axis, the Worse absolute value as the vertical axis, and the mean value acting as the critical line, in order to help the user understand the importance of protective clothing needs. As illustrated in Fig 2, the four-quadrant model diagram can depict the distribution of each demand, capture its relevance and urgency, and categorize the functional qualities based on the data [32].

According to the KANO model, the functional requirements of protective clothing are classified into four quadrant diagrams to identify the important requirements to be considered in the design process [33]. The Better values of C24, C23, C19, C8, C12, C13, C21 and C3 have a significant impact on the satisfaction level, where C24 is "convenient for storing and carrying objects". C24 is the "convenience of storing items". C23 is for ’Convenience of storage’, which is the convenience of storing and retrieving communication tools in the clothing for the various departments that need to communicate with each other during epidemic prevention work. C19 is for ’reasonable colour differentiation’. In the case of an epidemic, the colour of the clothing is used to differentiate between different departments of epidemiologists in the event of a concentration of personnel, so that they can distinguish between doctors, nurses, government personnel and volunteers. For ’seasonal wear’, the need to provide breathable and cool comfort features is necessary in the summer when working in closed garments makes it difficult for epidemiologists to withstand the heat. For example, the structural relationship between the interior and exterior of the closed lapels is designed to take into account both the tightness of the protection and the suitability for the season. C12 is the ’modular function’, where the protective clothing is designed to be modular in composition and the overall appearance of the protective clothing is designed to be made up of independent modular components, each of which can be dismantled, disinfected and recycled to enhance resource utilisation in special medical situations [34]. C13 is "softness of materials", which means that protective clothing should be made of soft materials and have an elastic waist to meet the needs of different body types and bring comfort to the user. "C21 is "no burden on the user when putting on and taking off protective clothing", which means that putting on and taking off protective clothing during epidemic prevention work will not burden the user, taking into account the characteristics of one-piece and split protective clothing and the elastic design of the head hood to reduce the wearing process and cognitive load. The waterproof function of the garment is a key feature of the suit.

Through the results of the demand attribute division of the KANO model of protective clothing design in the previous stage, focus on the examination of C24, C23, C19, C8, C12, C13, C21 and C3 demand elements, and calculate the influence weights of these eight key demand elements ω_i_, the calculation steps are as follows [35]:

$$\omega_{i}=\text{max}\left\{\frac{S_{i}}{\sum S_{i}},\left|\frac{D_{i}}{\sum D_{i}}\right|\right\}$$

The impact of the Kano demand analysis was calculated based on the above formula and the results are shown in Table 6.

**Table 6:** Impact results of the Kano demand analysis (Source: Author’s own research).

Demand	*S*_*i*_	*D*_*i*_	*ω*_*i*_
C24	0.743	-0.422	0.135
C23	0.734	-0.569	0.133
C19	0.697	-0.459	0.127
C8	0.701	-0.467	0.127
C12	0.688	-0.606	0.139
C13	0.615	-0.596	0.137
C21	0.651	-0.578	0.133
C3	0.681	-0.660	0.151

### Analysis of protective clothing design elements based on QFD models

The QFD model is a multi-level deductive analysis method that translates user requirements into design elements, quantifies user needs and finds key technical objectives, solves the "what to design" problem and provides a precise decision direction for design. Quality (HoQ) to achieve the expectation of meeting user requirements. Quality planning for user requirements consists of a competitive market assessment and quality target planning. Through research and analysis of relevant protective clothing in the medical industry, the design priorities and quality averages of existing epidemic-proofing products are clarified. The main objective is to complete a quantitative analysis of the quality elements of protective clothing, which is the basis of the importance of the QFD method used to assess users [36]. By analysing the present value of the demand factor *Q*_*a*_ and the target value of the demand factor *Q*_*b*_, it is possible to calculate the demand factor improvement rate *R*_*i*_ with the following formula:

$$R_{i}=\frac{Q_{b}}{Q_{a}}$$

In order to ensure the professionalism of the questionnaire assessment, the research subjects were protective clothing designers, hospital doctors and nursing staff. A five-point Liker scale scoring method with scores of (1, 2, 3, 4, 5) corresponding to the levels (poor, fair, medium, good, excellent) was used to evaluate the demand factor indicators [37], resulting in a demand factor improvement rate *R*_*i*_, as shown in the Table 7.

**Table 7:** Quality planning table for demand elements (Source: Author’s own research).

Elements of demand	Competitive evaluation			*Q*_*a*_	*Q*_*b*_	*R*_*i*_
	H	J	P			
C24	2	3	2	3	3	1
C23	1	2	2	2	3	1.5
C19	4	3	1	4	4	1
C8	3	2	2	3	4	1.33
C12	4	4	3	3	4	1.33
C13	3	1	2	2	3	1.5
C21	2	2	3	3	3	1
C3	3	4	2	3	3	1

The main purpose of building a quality house with epidemic-proof clothing design is to achieve mapping and conversion from user requirement elements to design function elements. The tool contains elements that measure the degree of user expectations and needs for product functions, so as to obtain a ranking of design elements to control the quality of product design and improve the user experience of protective clothing in the epidemic-proofing process [38]. From the above analysis of the importance of user requirements for protective clothing, it was found that the main requirements are divided into six areas: safety requirements, operational requirements, performance requirements, comfort requirements, identification requirements and environmental requirements. The needs are translated into ten design elements of the protective clothing, such as S_1_ for liquid resistance, S_2_ for germ effect, S_3_ for functional storage, S_4_ for ease of operation, S_5_ for airtightness of fit, S_6_ for lightness, S_7_ for comfort of materials, S_8_ for visualisation, S_9_ for environmental protection, S_10_ for colour recognition, S_11_ for shape coordination, S_12_ for Emotional design, S_13_ for recycling. The design objectives of the anti-disease clothing were unfolded and a scale of 0, 1, 2, 3, 4 and 5 was used to indicate no relevance at all, weak relevance, medium relevance and high relevance, respectively [39], to determine the degree of relevance between user needs and design elements. The requirement element-design element matrix *C*S*ij* was constructed (the data are shown in Table 8 *Ci* and S*j* crossed), and the requirement importance N*i* and its relative weight N′*i* and design function importance *Ej* and its relative importance were calculated respectively *E*′*j*, which were calculated according to the following equation [40]:

$$N_{i}=\frac{\omega_{i}}{R_{i}}$$

$${N^{\prime }}_{i}=\frac{N_{i}}{\sum \;N_{i}}$$

$$E_{j}=\sum_{i=1}{N^{\prime }}_{i}CS_{ij}$$

$${E^{\prime }}_{j}=\frac{E_{j}}{\sum E_{j}}$$

**Table 8:** Protective clothing design build quality house (Source: Author’s own research).

Demand elements	Design functional elements													N*i*	*N′i*
	*S*_*1*_	*S*_*2*_	*S*_*3*_	*S*_*4*_	*S*_*5*_	*S*_*6*_	*S*_*7*_	*S*_*8*_	*S*_*9*_	*S*_*10*_	*S*_*11*_	*S*_*12*_	*S*_*13*_		
C24	4	2	0	0	1	0	1	0	0	3	0	0	0	0.135	14.6%
C23	3	5	3	2	0	0	1	0	0	4	1	0	0	0.089	9.6%
C19	3	5	2	1	1	0	0	4	0	0	0	1	2	0.127	13.7%
C8	1	4	0	5	0	0	1	0	0	0	1	0	0	0.095	10.3%
C12	2	0	4	0	3	5	0	3	0	0	0	3	0	0.104	11.3%
C13	0	3	3	0	5	1	0	0	1	0	2	0	3	0.091	9.9%
C21	3	5	0	5	0	0	0	0	1	1	0	0	0	0.133	14.3%
C3	1	0	3	3	0	1	0	0	1	0	0	0	1	0.151	16.4%
*Ej*	220.4	288.1	179.9	205.2	111.4	82.6	34.5	88.5	40.6	96.5	39.6	47.5	73.3		
*E*′j	14.6%	19.1%	11.9%	13.6%	7.4%	5.5%	2.3%	5.9%	2.7%	6.4%	2.6%	3.1%	4.9%		
Sort by	2	1	4	3	5	8	13	7	11	6	12	10	9		

Based on the above steps, the design quality house of the epidemic-proof clothing is constructed and from this the importance ranking of the design functional elements of the epidemic-proof clothing can be obtained (e.g. Table 8) [41].

After completing the analysis of protective clothing design elements based on the QFD model, the mapping relationship between user needs and design elements has been clarified. Next, specific design proposals need to be developed based on these analysis results. By converting the importance and improvement rate of the need elements into the weight of the design functions, we ensure that each design proposal effectively meets the core needs of users. The following section will utilize the PUGH matrix method to comprehensively evaluate multiple design proposals and select the optimal one.

### Comprehensive evaluation of PUGH-based protective clothing design solutions

**Fig 3:**
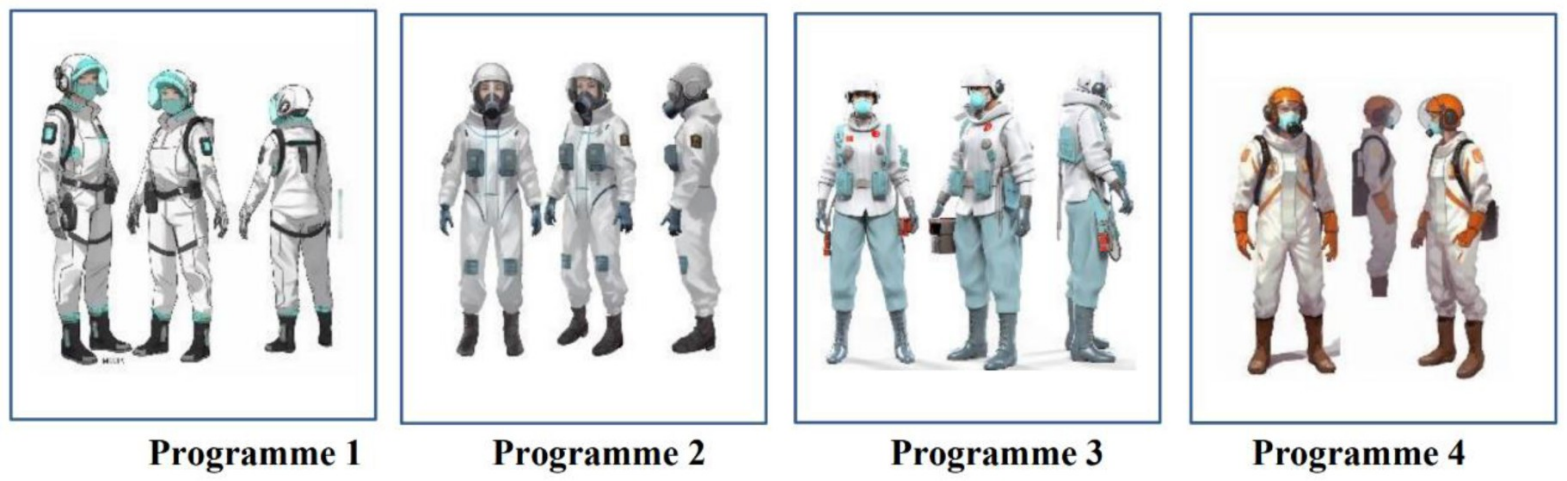
Diagram of the four conceptual design options (Drawn by the author).

For the design solution trade-off process during the product development process, the PUGH matrix selection method offers a scientific decision-making tool [42]. It helps designers evaluate and rate several design ideas in order to choose the best design concept to satisfy the design criteria of the requirement metrics. The design team conducted an internal evaluation and developed four different concept designs based on the analysis of design requirements and design functional elements, combined with the usage scenarios of user experience, based on the QFD Quality House’s ranking analysis of the importance of functional requirements (As shown in Fig 3).

Firstly, according to the design functional elements and weights analysed in the QFD, the design team identified Scheme 1 as the benchmark scheme from the schemes to be evaluated, and set all the grades to 3. 5 scale (1 means very poor, 2 means slightly poor, 3 means same, 4 means slightly better, 5 means better); finally, each design function element was graded from S1-S13, and each item was compared with the benchmark 1 scheme according to the evaluation criteria, calculated from the size of the score combined with the importance of the design criteria [43], and calculated the overall score of the design scheme, the calculation results are shown in Table 9 shown in the following formula [44]:

The jth design function element indicator score for the kth option *F*_*jk*_ = *E*′_*j*_ ⋅ *d*_*jk*_.

**Table 9:** Overall rating for protective clothing design (Source: Author’s own research).

Indicators	Weighting	Option 1		Option 2		Option 3		Option 4	
		Grade	Score	Grade	Score	Grade	Score	Grade	Score
*S*_*1*_	14.6%	3	0.438	4	0.585	4	0.585	4	0.585
*S*_*2*_	19.1%	3	0.573	4	0.764	4	0.764	3	0.573
*S*_*3*_	11.9%	3	0.358	3	0.358	3	0.358	3	0.358
*S*_*4*_	13.6%	3	0.408	2	0.272	3	0.408	2	0.272
*S*_*5*_	7.4%	3	0.222	3	0.222	4	0.295	2	0.148
*S*_*6*_	5.5%	3	0.164	2	0.110	3	0.164	3	0.164
*S*_*7*_	2.3%	3	0.069	3	0.069	4	0.091	2	0.046
*S*_*8*_	5.9%	3	0.176	3	0.176	3	0.176	2	0.117
*S*_*9*_	2.7%	3	0.081	2	0.054	3	0.081	3	0.081
*S*_*10*_	6.4%	3	0.192	3	0.192	2	0.128	2	0.128
*S*_*11*_	2.6%	3	0.079	3	0.079	4	0.105	4	0.105
*S*_*12*_	3.1%	3	0.094	2	0.063	2	0.063	1	0.031
*S*_*13*_	4.9%	3	0.146	2	0.097	3	0.146	3	0.146
**Overall rating of the programme**		3.000		3.039		3.365		2.754	

Calculate the overall programme score *F*_*k*_ = Σ_*j*_ *F*_*jk*_.

The ranking of the design concept solutions for the epidemic-proof clothing is solution 3 > solution 2 > solution 1 > solution 4, with solution 3 being the best design solution and being the most in line with the design project development requirements in terms of comprehensive indicators, as shown by the results of the comprehensive scoring in Table 9. The final choice was closer to the optimal design solution after the reasonableness, benefits, and drawbacks of the available solutions were assessed using the PUGH matrix selection method [45]. It satisfies the anticipated outcomes of the design concept, effectively addresses the current user requirements for medical protective apparel, and reflects the opinions of the majority of healthcare experts.

## Results

**Fig 4:**
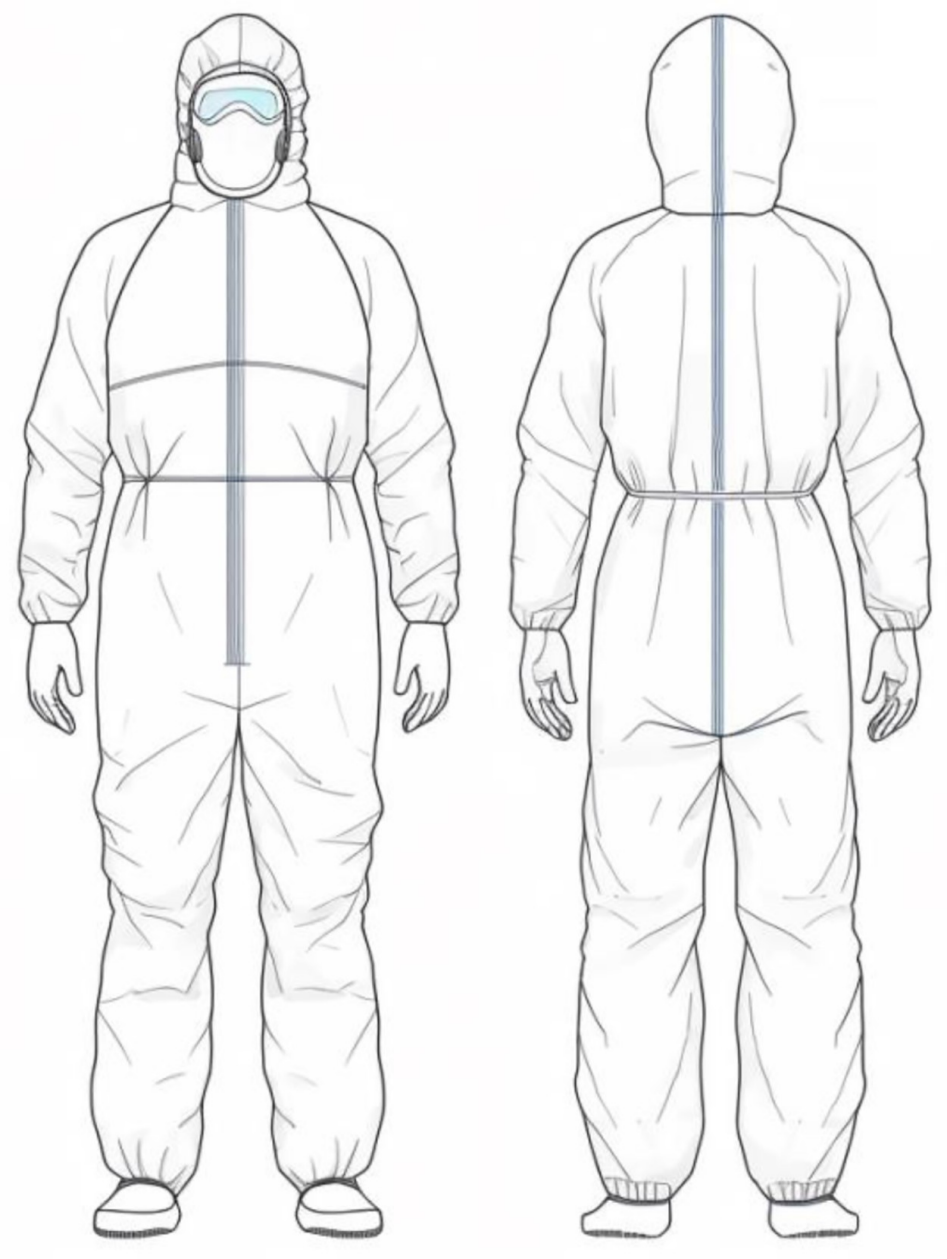
Original protective clothing design (Source web).

**Fig 5:**
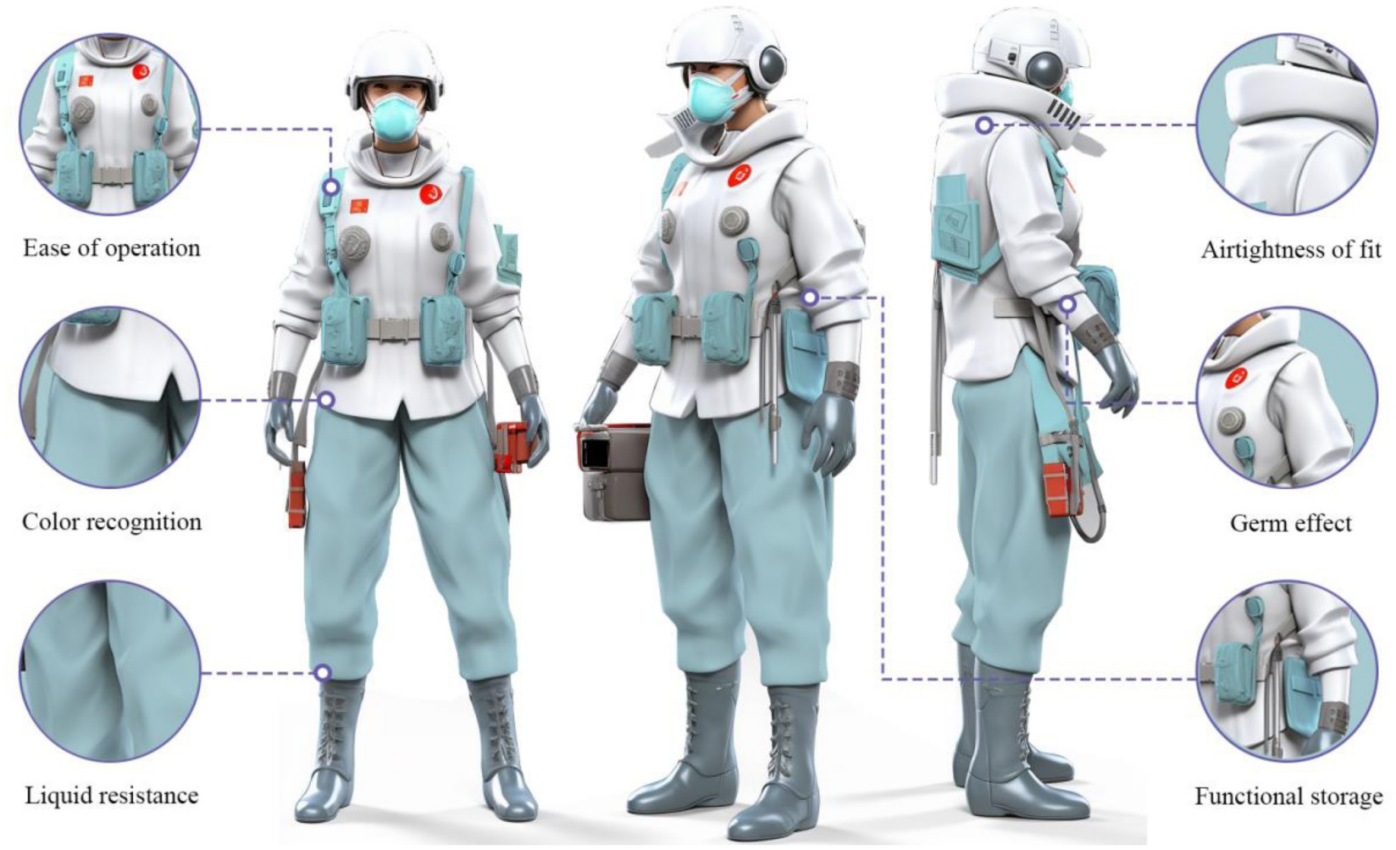
Conceptual design of medical protective clothing based on user needs (Drawn by the author).

Designs of protective clothing that are currently available on the market were chosen for comparison with the preferred design concept solution in order to determine whether the preferred protective clothing concept design solution satisfies the audience’s needs 3. The approach for the protective clothing design was compared to the original design sample and concept design option 3 in order to assess its validity and viability [46]. The results are depicted in Figs 4 and 5.

**Fig 6:**
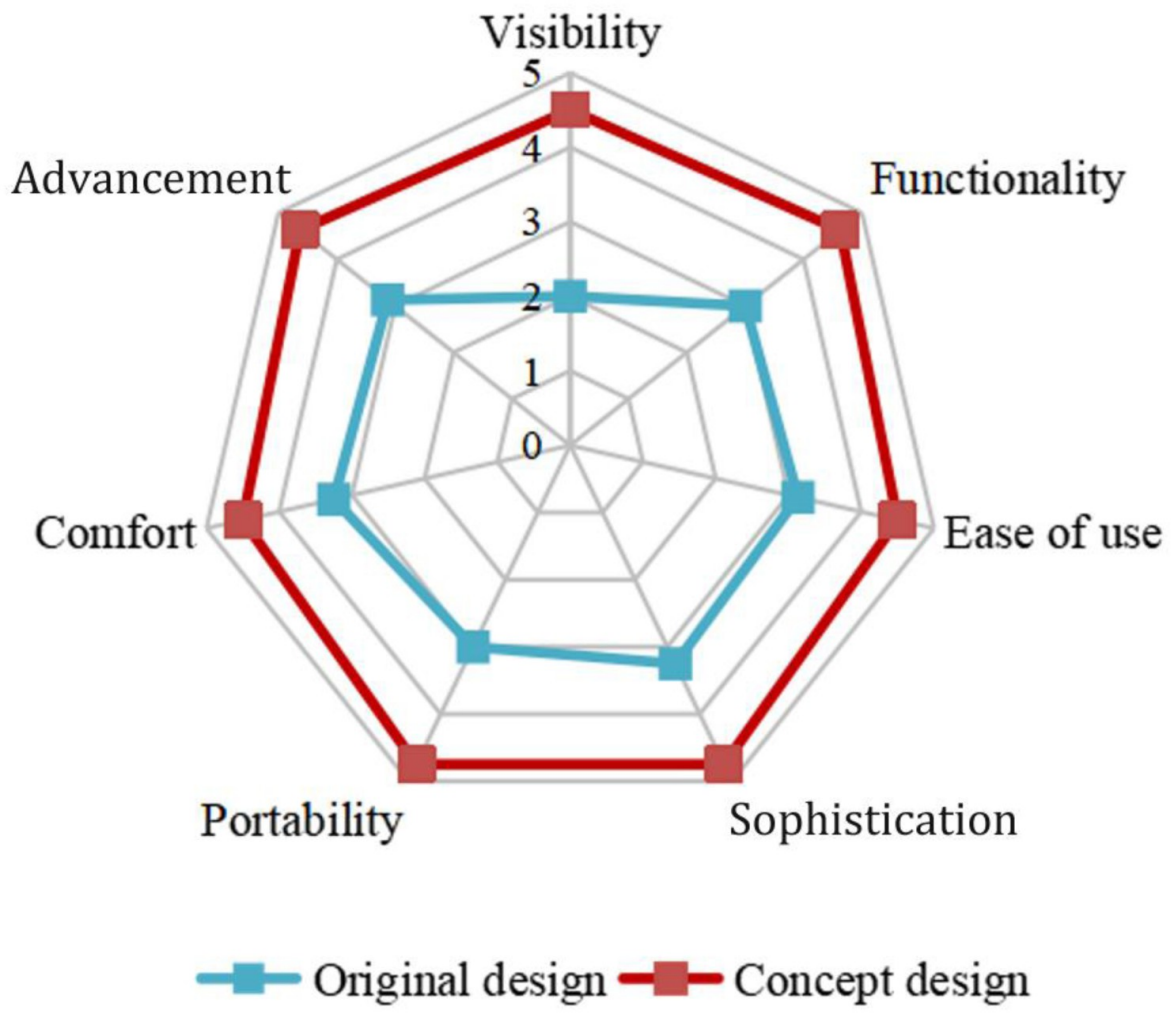
Radar diagram of medical protective clothing based on user needs.

A five-point scale was used in a questionnaire survey to gauge audience satisfaction with the necessity of protective equipment. The study was assessed using a validated user experience scale to confirm the scientific validity of the design [47]. Indicators including visibility, functionality, ease of use, sophistication, portability, comfort, and advancement were utilized to examine the validity of the research proposal. By employing Questionnaire Star’s online research technique for healthcare professionals, a total of 500 questionnaires were distributed, and 483 valid surveys were returned. The findings (Table 10, Fig 6) indicate that while the audience satisfaction score for the user needs-based medical protective apparel design concept was 3.65, the average score for the features of the original design sample was 3.15. It is clear that the medical protective clothing created with the aid of this research methodology can, to a certain extent, increase audience satisfaction, which serves as a guiding principle for the creation and design of protective clothing.

**Table 10:** Changes in design satisfaction for medical protective clothing (Source: Adapted from Wang Jun et al., 2023).

Properties	Original design	Concept design
Visibility	2	4.5
Functionality	3	4.63
Ease of use	3.13	4.5
Sophistication	3.25	4.75
Portability	3	4.75
Comfort	3.25	4.5
Advancement	3.13	4.63

## Discussion

Overall, the Kano-QFD-PUGH method is a commonly used product design analysis method that is important in helping in the conceptual design phase of protective clothing [48]. Firstly, the Kano model effectively helps the design team to identify user requirements and helps to ensure that design elements meet user expectations and needs; secondly, the use of the PUGH matrix for evaluation and optimisation helps to provide quality and performance of protective clothing; finally, the design team can identify and solve problems during the design phase, thus reducing the cost of fixes and improvements at a later stage, and the use of the PUGH matrix for evaluation which helps to improve design efficiency and reduce design cycle times. The Kano-QFD-PUGH method is used to study the development of design practices for protective clothing and to propose an integrated approach to understanding the functional requirements of protective clothing design from the perspective of user needs [49]. Protective clothing design is a comprehensive system project and the focus of this study is on the pre-conceptual design phase, with no involvement in the post-protective clothing performance testing. The method provides a preliminary theoretical basis for the design and development of medical protective clothing, offers the possibility of realising scientific and precise design requirements, and helps and promotes the optimisation and upgrading of protective clothing at a later stage.

However, this method has some shortcomings in the research. The application of the Kano-QFD-PUGH method to the design of protective clothing requires the consideration of a variety of factors, such as the influence of chemical and physical properties on protective clothing [50]. There is a complex interrelationship between these factors that needs to be considered in an integrated manner. In later studies, the protective properties of protective clothing will be tested, and the materials, accessories and processes of protective clothing will be studied to further optimise the physical properties of protective clothing and to achieve functional, lightweight and intelligent development of protective clothing, thus improving the efficiency of epidemic prevention work and humane service [51].

## Conclusion

The Kano-QFD-PUGH method is a design method that combines customer requirements, quality function development and the PUGH method. Its innovation lies in the organic combination of the different methods to achieve a more comprehensive and systematic product design. In the post-epidemic era the need for protective clothing is not going to go away and it is becoming an essential medical tool for healthcare workers [52]. In the midst of an epidemic, awareness of disease prevention and control has been raised. The design of protective clothing can make the public more aware of the ways in which diseases are transmitted and the preventive measures that can be taken, thus increasing the public’s awareness of disease prevention. In order to adapt to the development of the times and to prevent future infectious diseases, the focus on the functional needs of protective clothing has increased. Protective clothing design should solve the problem from the essence, not only pay attention to the innovation in the function of medical clothing, but also focus on the rationalisation and science of the design transformation and the optimisation of the development process, so as to effectively improve the level of comprehensive satisfaction of users for medical clothing [53]. Based on the summary of the research on the design of protective clothing, the main findings of this study are as follows:

1. Through a theoretical study of the design and development of protective clothing, the Kano-QFD-PUGH method is effectively applied to the design of medical protective clothing, which can meet user needs, improve design and development efficiency and quality, provide a complete design process for protective clothing design and development, and provide theoretical value for protective clothing design and development.
2. The use of the Kano-QFD-PUGH integrated product development process provides a scientific approach to protective clothing design and development. It breaks with the traditional design principles of protective clothing developers, which are centred on the subjective consciousness of the designer. Traditional design developers tend to focus solely on the exploration of user requirements, without weighting design elements in, and make their own subjective judgements in the design determination phase. This method solves the problems associated with "what needs", "what to design" and "which design is good". It helps designers to use the method to extract design requirements accurately, to clarify development priorities and to scientifically select design solutions, improving the designer’s design development capability and efficiency.
3. The method has been validated through design practice by means of a questionnaire survey of the final design concept solution, and from the results a good user evaluation result was achieved. This provides a practical example for the development of protective clothing and can help designers to better understand and apply the method. It provides a reference for the design, development and production of related medical products in the future.

## Supporting information

S1 File This document contains quantitative research data related to the design of protective clothing. (XLSX)
